# Diet dichotomy between two migrant seabirds breeding near a high Arctic polynya

**DOI:** 10.1098/rsos.160982

**Published:** 2017-03-22

**Authors:** Isabeau Pratte, Kelly A. Boadway, Shanti E. Davis, Mark Maftei, Mark L. Mallory

**Affiliations:** 1 Department of Biology, Acadia University, 15 University Drive, Wolfville, Nova Scotia, CanadaB4P 2R6; 2 High Arctic Gull Research Group, 109 Kings Road, Bamfield, British Columbia, CanadaV0R 1B0

**Keywords:** niche segregation, stable isotopes, Sabine's gull, Arctic tern, incubation

## Abstract

High Arctic polynyas are predictable areas of open water, which offer long-distance migrant seabirds a reliable source of food during a period when they have to replenish and accumulate energy for reproduction. Investigating the interaction between species nesting sympatrically in the vicinity of polynyas should provide insights into the role that such oceanographic features play for pre-breeding seabirds. We used stable isotopes (δ^13^C and δ^15^N) to compare the diet of two ground-nesting seabirds, Sabine's gull (*Xema sabini*) and Arctic tern (*Sterna paradisaea*), nesting on an island adjacent to a recurring polynya in the Canadian high Arctic in 2008 and 2009. We show that, unlike Arctic terns, the diet of Sabine's gulls appears to include a non-negligible amount of terrestrially derived prey during early incubation, and that overall both species segregate their dietary niche during pre-laying and early incubation.

## 1. Introduction

For long-distance migrants, the optimal timing of arrival at breeding sites must closely match favourable conditions for initiating reproduction [1]; however, environmental cues influencing migration may not accurately predict conditions at the nesting location [2]. Such a climatic and/or resource mismatch could constrain individuals upon their arrival and delay the onset of reproductive activities [3]. Thus, the selection of breeding sites near areas with predictable resources could confer an advantage to individuals, especially those breeding in generally barren habitats such as the high Arctic, a region in which the time available for breeding is extremely constrained, and where spring snow cover has a marked effect on annual variation in peak food abundance and timing [4,5] as well as nest site availability [6].

Polynyas in the Canadian Arctic Archipelago are kept free of ice by a combination of tidal currents, wind and upwelling, factors which also contribute to making these areas highly productive [7,8]. For Arctic seabirds, polynyas near suitable breeding sites can provide predictable resources during the pre-breeding and breeding season as they provide reliable access to open water within regions otherwise still covered by pack ice [7–9]. Given the persistence of sea ice throughout the summer in the Canadian high Arctic [10], polynyas are probably key features which led to the colonization of this region by seabirds [11,12]. However, high inter-annual variation in the extent of open water early in the season at some polynyas could limit access to marine resources, and ultimately cause or enhance intra- and interspecific competition among seabirds nesting near these sites.

Many Arctic seabirds are primarily income breeders (e.g. *Fulmarus glacialis* [13]; *Sterna paradisaea* [14]) relying on exogenous nutrients for egg formation. These species replenish energetic and nutrient reserves on their breeding grounds after their northward migration, and as such rely on local foraging opportunities. The availability of resources upon arrival at breeding sites will thus have a major influence on an individual's capacity to reproduce, the timing of breeding and on their relative investment [15–17]. Investigating the interaction between species nesting sympatrically near polynyas would thus be expected to provide insights into the role of these oceanographic features in attracting and sustaining colonies of seabirds in the high Arctic.

We compared the diet of two ground-nesting seabirds, Sabine's gulls (*Xema sabini*) and Arctic terns (*Sterna paradisaea*), on a small island adjacent to a recurring polynya in the Canadian high Arctic Archipelago. Both species are highly pelagic, trans-equatorial migrants and are approaching the northern extent of their range at this site. A previous study of these species at lower latitudes [18] suggested that they overlapped in diet during the pre-breeding season, but shifted towards foraging in different areas and presumably on different prey as the season progressed. The study area used in that project [18] represented typical breeding habitat for both Sabine's gulls and Arctic terns across most of their range in the Canadian Arctic: coastal low Arctic tundra with extensive freshwater wetlands and ponds in which birds forage almost exclusively terrestrially during the breeding season. At our study site, 1500 km to the north, birds nest on a small gravel island which is typically surrounded by extensive sea ice for hundreds of kilometres in all directions, except for a small polynya of about 10 km^2^ which occurs immediately adjacent to the island [12]. Consequently, we expected that marine resources available to these species would be limited to those available in the polynya. We predicted: (i) both species would rely heavily on marine prey accessible within the polynya, and (ii) that the species would select different prey to minimize interspecific competition. To test these predictions, we examined stable isotope values (δ^13^C and δ^15^N) in blood plasma samples from both Sabine's gull and the Arctic tern to determine if they differed in their isotopic niche at this high Arctic colony.

## 2. Material and methods

Nasaruvaalik Island, Nunavut is located in the Canadian high Arctic (75°49′ N, 96°18′ W) and supports the largest and most diverse colony of ground-nesting seabirds in the region [12]. Arctic terns are the most numerous seabirds at this site (approx. 500 nests) and Sabine's gulls (approx. 30 nests) breed within the tern colony extent. Both species winter in the Southern Hemisphere [19–22] and are income breeders [14], relying on resources obtained upon arrival at the breeding site to perform breeding and pair-bonding displays and initiate egg-laying.

To investigate adult diet during the pre-laying and incubation periods, we used two approaches. First, we sampled blood of individual birds during incubation, and centrifuged each sample to separate the blood cells from the plasma, keeping the latter since this tissue reflects diet in the previous 2–7 days [23,24]. In 2008, sampling was done on the 7–15 July for Sabine's gull (*n* = 18) and 15–28 July for the Arctic tern (*n* = 10); while in 2009 both species were sampled on the 11–18 July (Sabine's gull, *n* = 20; Arctic tern, *n* = 24). Birds were captured during incubation using a bow-net trap, and their eggs were removed and kept warm while dummy eggs were placed in the nest. If a bird took more than 30 min to resettle on its nest, we abandoned the capture attempt, so the bird could resume incubation. Second, during 2008–2011 we collected prey brought back to the nest site by breeding birds. In July 2011, we also sampled other putative prey items in the marine and terrestrial environments using aquatic D-frame nets to sweep through the water column, or by picking up individuals on the land (table 1; see also [26]). We used prey samples as references for further interpretation of the bird isotopic values, although we were unable to collect all prey known to be consumed by the two species. In 2008, the first egg was found on 25 June for Sabine's gull and 28 June for the Arctic tern, and 30 June for both species in 2009. Sabine's gull and the Arctic tern were feeding in the polynya when researchers arrived each year (16 June 2008 and 17 June 2009). Note that this work was a subset of a larger project on the breeding biology of both species at high latitudes [27,28].

**Table 1.** Mean ± s.d. of plasma δ^13^C and δ^15^N values of Sabine's gull and Arctic terns in 2008 and 2009, as well as of different food items from the marine and terrestrial food webs collected at Nasaruvaalik Island, Nunavut. Not all possible food items present in the diet of the species were collected.

		*n*	δ^13^C (‰)^a^	δ^15^N (‰)
2008	Sabine's gull	18	−17.53 ± 2.04	13.52 ± 1.76
	Arctic tern	10	−17.03 ± 0.64	14.71 ± 0.38
2009	Sabine's gull	20	−23.22 ± 0.93	8.84 ± 0.71
	Arctic tern	22	−15.89 ± 0.89	13.72 ± 1.04
marine food web^b^				
	harpacticoid copepod	8	−17.23 ± 1.56	5.97 ± 0.46
	calanoid copepod	8	−15.34 ± 3.66	9.92 ± 1.08
	amphipod^c^	19	−16.33 ± 2.85	10.41 ± 2.09
	Arctic cod (*Boreogadus saida*)	16	−20.17 ± 1.36	13.51 ± 0.61
terrestrial food web^d^				
	saxifrage	3	−29.73 ± 1.08	1.55 ± 0.29
	lichen	10	−25.15 ± 1.94	−0.25 ± 2.48
	terrestrial invertebrate^d^	22	−26.73 ± 1.08	6.12 ± 2.15
	moth	4	−28.75 ± 0.71	13.40 ± 2.06

^a^The bird and marine food web δ^13^C values were normalized for lipid content [25] except for Arctic cod samples, which were lipid-extracted.

^b^Items were collected in 2008, 2009 and 2011 at Nasaruvaalik Island.

^c^Amphipod includes species from the genus *Gammarus*, *Gammaracanthus* and *Themisto.*

^d^Terrestrial invertebrate includes chironomids, collembolla and other flies; moths were separated from that group because of their surprisingly high δ^15^N values.

Blood was collected by pricking the brachial vein with a 27-gauge needle and collecting approximately 150 µl of blood in heparinized capillary tubes [29]. Within 5 h, the blood was spun in a microhaematocrit centrifuge for 5 min to separate the plasma from the blood cells [25], and the plasma was then frozen at −20°C until analysis. Tissue samples (plasma and prey) were analysed by the Stable Isotopes in Nature Laboratory (SINLAB) at the University of New Brunswick using well-established procedures. Briefly, samples were combusted in an elemental analyser (NC2500), and gases were sent to the isotope-ratio mass spectrometer (Delta Plus/Conflo II) using a continuous flow interface. Data are reported as differences in isotopic ratios, for which the units are parts per mil (‰) compared with Vienna-Pee Dee Belemnite (V-PDB) for carbon, and atmospheric nitrogen (AIR) for nitrogen. Procedural details are outlined in English *et al.* [30]. During analyses, three secondary standards were run by SINLAB: nicotinamide (δ^15^N: −1.78‰, s.d. ± 0.01; δ^13^C: −34.22 ± 0.074‰, *n* = 3), bone liver standard (δ^15^N: 7.25 ± 0.18‰; δ^13^C: −18.68 ± 0.03‰, *n* = 3) and muskellunge (*Esox masquinongy*) muscle standard (δ^15^N: 12.43 ± 0.07‰; δ^13^C: −23.30 ± 0.01‰, *n* = 3). Check standards were also run to assess analytical accuracy: acetanilide (δ^15^N: −2.06 ± 0.11‰; δ^13^C: −27.65 ± 0.05‰, *n* = 5), N_2_ (δ^15^N: 20.58‰, *n* = 1) and CH_7_ (δ^13^C −31.84‰, *n* = 1). Arctic cod (*Boreogadus saida*) muscles were lipid-extracted; however, no lipids were extracted from our plasma samples, and thus we used the method suggested by Post *et al.* [31] to normalize δ^13^C for lipid content of the birds and other marine prey sources:

$$\delta^{13}{\text{C}}_{\mathrm{normalized}}=\delta^{13}{\text{C}}_{\mathrm{untreated}}-3.32+0.99\times \text{C}:\text{N.}$$

**Figure 1.**
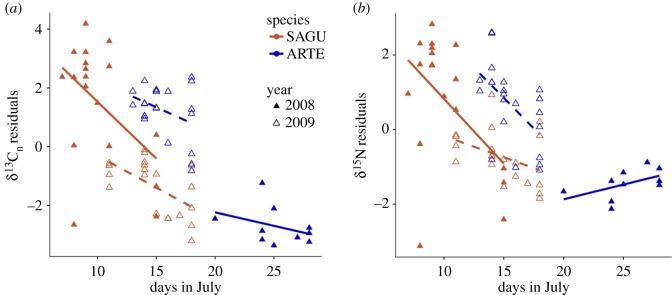
Relationship between date sampled and the residuals extracted from GLM models with only species and year as fixed effects to show the significant influence of date sampled on the variation in (*a*) δ^13^C_n_ and (*b*) δ^15^N for Sabine's gull (SAGU; orange) and Arctic tern (ARTE; blue) in 2008 (filled) and 2009 (open) at Nasaruvaalik Island. Trend lines represent the best linear fit to visually display the direction of the relationship for δ^13^C_n_ and δ^15^N for each year and species.

We used multivariate analysis of variance (MANOVA) to test the effect of the factors ‘species’ and ‘years’ as well as the linear covariate ‘date sampled’ (days in July) and their interactions on both normalized δ^13^C (δ^13^C_n_) and δ^15^N. Individuals were sampled early in the breeding season but on different days, and their isotopic value could have represented food ingested at their arrival at the breeding colony. Accordingly, we incorporated sampling date in our models. Half-life of carbon and nitrogen in blood plasma for small seabirds is less than 7 days [23], although it takes more than twice that long to reach isotopic equilibrium after a switch in diet [32]. If differences were found following the MANOVA model (*p* ≤ 0.05), we used a generalized linear model (GLM, family = Gaussian) approach to test, with *F*-statistic, the effect of the explanatory variables mentioned above and their interaction on each response variable (δ^13^C_n_ and δ^15^N). We selected the best model using Akaike's information criterion for small sample size (AICc). Based on AICc model selection (electronic supplementary material, S1), the top three models were equivalent (ΔAIC < 2.0) at explaining variation in δ^13^C_n_, but we chose to present the third model that tested for the effect of ‘species’, ‘year’ and ‘date sampled’ along with the interactions between ‘year’ and ‘species’, and between ‘date sampled’ and ‘species’. For δ^15^N, we kept the model that included all of the fixed effects and their interaction (electronic supplementary material, S1). To represent the influence of date sampled on δ^13^C_n_ and δ^15^N, we used the residuals of the GLM model without the effect of date sampled, and plotted those residuals against date (figure 1). We used stable isotopes Bayesian ellipses in R (SIBER) from the package ‘SIBER’ [33] and used a probabilistic method [33] to assess if differences in isotopic niche width (‰^2^) between species each year and within species between years were significant following the estimated *a posteriori* distribution of Bayesian-simulated standard ellipse areas (SEA_b_).

## 3. Results

**Figure 2.**
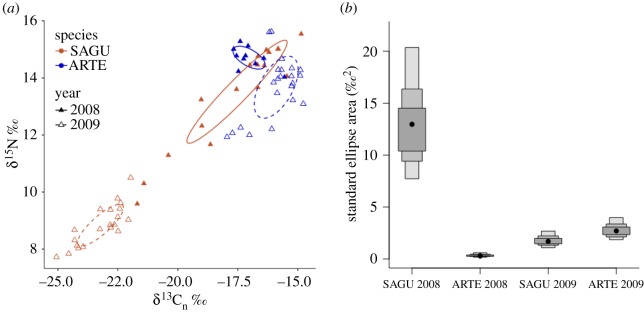
Isotopic niche area based on δ^13^C_n_ and δ^15^N values in the plasma of adult Sabine's gulls (SAGU) and Arctic terns (ARTE) sampled during incubation at Nasaruvaalik Island, Nunavut in 2008 and 2009. (*a*) Standard ellipses (40% credible interval, [30]) of the individuals are represented. (*b*) Density plots showing the mean ellipse areas (black dot) and their credible intervals (50%, 75% and 95%) obtained following a Bayesian approach of posterior estimate of simulated standard ellipse areas (SEA_b_; [30]).

Bird species, year, date sampled and their interaction were significant in explaining variation in δ^13^C_n_ and δ^15^N values in breeding gulls and terns (MANOVA; *F*_7,62_ > 5.72, *p* < 0.005). Only in 2009, Sabine's gulls had lower δ^13^C_n_ than Arctic terns (table 1; GLM; *F*_5,69_ = 9.96, *p* = 0.002; figure 2*a*), and gull δ^13^C_n_ values overlapped with that of the values in terrestrial-based prey (table 1). For both species, individual birds sampled later generally had lower δ^13^C_n_ (GLM; *F*_5,69_ = 146.08, *p* < 0.001; figure 1), which indicated a rapid turnover rate of carbon following arrival on the breeding ground and a switch in diet upon arrival at the breeding colony (figure 1). The interaction between ‘date sampled’ and ‘species’ was not significant at explaining variation in δ^13^C_n_ (*F*_5,69_ = 1.58, *p* = 0.22). Arctic terns had a diet more enriched in nitrogen than Sabine's gull, and in 2008, both species foraged at a higher trophic level than in 2009 (table 1), a tendency that seemed mostly driven by the low δ^15^N values of Sabine's gull in 2009, which could be the result of different sampling dates between years, resulting in the significance of the three-way interaction terms (figures 1*b* and 2*a*; GLM; *F*_7,69_ = 7.99, *p* = 0.006).

Based on the probabilistic method of the Bayesian-simulated ellipses [33], the isotopic niche width varied across species and years. Sabine's gulls had a much larger niche width (SEA_b_ 12.07‰^2^) than Arctic terns (0.38‰^2^, *p* < 0.001) in 2008 (figure 2*b*). Both species had similar niche width in 2009, but were clearly segregated (SEA_b_ of 1.75‰^2^ and 2.49‰^2^, respectively; figure 2*a,b*). Sabine's gull niche width was larger in 2008 than 2009 (*p* < 0.001), while the opposite was observed for Arctic terns (*p* < 0.001; figure 2*b*).

## 4. Discussion

As in other polynyas in the Canadian Arctic, the recurrent open water surrounding Nasaruvaalik Island appears to provide a predictable foraging environment for long-distance migratory seabirds upon their arrival at their breeding site. However, the total area of open water of the polynya is quite restricted in late June when the birds arrive (estimated 5 × 2 km; sufficiently small to not be mapped in [8]). Although we regularly observed both bird species foraging in the polynya, stable isotope values (δ^13^C_n_ and δ^15^N) in the plasma of Sabine's gulls and Arctic terns over two breeding seasons revealed that Sabine's gulls have a broader isotopic niche than Arctic terns, and that gulls incorporate terrestrially derived prey into their diet, especially in 2009.

Our prediction that these two species would feed on different prey in the marine environment to reduce competition with each other was partially supported, but with the surprising result that Sabine's gulls were also exploiting non-marine prey, especially in 2009, while the elongated ellipse area suggested this also happened in 2008. Indeed, during several years of detailed study [27], we observed Sabine's gulls foraging in both marine and terrestrial environments annually, although we did not quantify observations nor time spent foraging in each environment. Nonetheless, based on those observations, we did not expect such a dramatic isotopic shift as we found. While higher δ^13^C_n_ and δ^15^N values in Arctic terns suggest that individuals were feeding at a high trophic level within the marine environment during the pre-laying and incubation periods, Sabine's gulls appeared to rely on different prey once they arrived at the colony; their δ^13^C_n_ and δ^15^N values were relatively similar to those of terns early in the breeding season (presumably reflecting the diet during migration or at arrival), but exhibited markedly lower values of both δ^13^C_n_ and δ^15^N once they were foraging locally. In fact, the low δ^13^C_n_ values of the gulls were consistent with the measured δ^13^C values of terrestrial prey at Nasaruvaalik Island (table 1), supporting observations of terrestrial invertebrates as important food sources for low Arctic Sabine's gull during the breeding season ([19,34–36]), and suggesting that even in the high Arctic, Sabine's gulls clearly exploit terrestrial prey resources if and when available.

For both species, we noted that δ^13^C_n_ values were strongly influenced by the date sampled. For Sabine's gull, this relationship suggests that conditions at arrival (snow cover, low abundance of insects) are not favourable to terrestrial feeding, thus that species might rely heavily on the polynya at that time. The decreasing trend in the δ^13^C_n_ values following date sampled, and the negative effect of date sampled by year on the δ^15^N values might also suggest a diet depleted in carbon and nitrogen at this high Arctic colony for Sabine's gull compared to the food consumed during recent migration, and that isotopic equilibrium was not yet attained upon the switch in diet after arrival at the breeding ground [37,38], especially in 2008 when Sabine's gull were sampled earlier. Sabine's gulls nesting at Nasaruvaalik Island spend the winter off the coast of Peru [21] and were recorded in the Beaufort Sea typically by 6 June on their spring migration north, reaching Nasaruvaalik Island by 9–25 June [39]. The isotopic values of marine invertebrates (i.e. euphausiids, *Neocalanus* sp., *Thysanoessa* sp.; credible food sources for migrating Sabine's gulls; [34]) in their staging and migration areas are around δ^13^C −19‰ and δ^15^N 11‰ [35–40], values that contrast with the high Arctic terrestrial and marine food webs of Nasaruvaalik Island (table 1).

Arctic terns in 2008 had a highly constrained isotopic niche, but were also sampled later in incubation, which suggested that they had reached the isotopic equilibrium associated with their breeding ground diet. In 2009, both species were sampled during the same period and their isotopic niche widths were clearly distinct and of similar size. We noted that 2009 followed a peak in abundance of lemmings (*Dicrostonyx* sp.), and large numbers of chironomids and other unidentified flies were observed coming out of the lemming burrows early in the season, which Sabine's gulls were observed eating. While terrestrial invertebrates are present every season, particularly high numbers associated with stochastic events such as peak lemming cycles are probably uncommon. Large numbers of lemmings have only been recorded in two of the last 10 years on Nasaruvaalik Island, but the appearance of this additional supplement to their diet during the pre-breeding season was clearly exploited by the gulls and not by the terns. This high terrestrial prey abundance could also have influenced the difference observed in δ^15^N and δ^13^C_n_ values of Sabine's gulls between 2008 and 2009, suggesting prey differences between years.

## 5. Conclusion

Together, our results corroborate the inclusion of terrestrial food sources in the diet of Sabine's gull, even at the northern limit of their known breeding range in a distinctly marine environment. Non-systematic observations in other years of data confirm that Sabine's gulls use terrestrial food sources each year at this latitude. We are unsure if invertebrates comprise as much of the diet as in 2009, a year of high terrestrial invertebrate productivity, although the pattern from 2008 suggested that a shift to predominantly terrestrial prey was underway during our sampling. While the proximity of a highly productive polynya makes Nasaruvaalik Island a particularly suitable nesting site for ground-nesting seabirds like Sabine's gulls and Arctic terns, the role of the numerically far more abundant Arctic terns as biovectors of nutrients [41] could also render the habitat within the tern colony particularly favourable for Sabine's gulls. Like many other seabirds, Arctic terns are important instruments of nutrient transport from the ocean to their colony, enhancing productivity of the terrestrial food web where they breed [41–45]. This nutrient enrichment is clearly visible in the high Arctic where tern colonies are much more vegetated than the barren surrounding environment [46] and thus potentially more attractive to Sabine's gulls [47]. We suggest that our results provide a novel interpretation of one of the potential factors leading to the well-noted but inconclusively explained nesting association between these two species in the high Arctic, which almost invariably occur together at higher latitudes [34].

## Supplementary Material

Supplementary material: Summary of AIC model selection explaining variation in plasma δ13Cn and δ15N values of Sabine's gulls and Arctic terns sampled during incubation at Nasaruvaalik Island, NU, in 2008 and 2009.

## Data accessibility

The data used for this study have been deposited in Dryad Digital Repository: (http://dx.doi.org/10.5061/dryad.n2d15) [48].
