# Supplementary material for: Diet dichotomy between two migrant seabirds breeding near a high Arctic polynya
Source: R Soc Open Sci. 2017 Mar 22;4(3):160982. doi: 10.1098/rsos.160982 (PMC5383845; doi:10.1098/rsos.160982)
Supplement: Supplementary material: Summary of AIC model selection explaining variation in plasma δ13Cn and δ15N values of Sabine's gulls and Arctic terns sampled during incubation at Nasaruvaalik Island, NU, in 2008 and 2009. [file rsos160982supp1.docx]

Supplementary material

S1. Summary of model selection explaining variation in plasma δ^13^C_n_ and δ^15^N values of Sabine’s gulls and Arctic terns sampled during incubation at Nasaruvaalik Island, NU, in 2008 and 2009. The effect of ‘species’, ‘year’, ‘date sampled’ and their interaction were tested. Only the top models with ∆AICc < 2.0 are presented. In bold are the selected models described in the *Results* section.

| **Model** | **AICc** | **∆ AICc** | **Weight** | **K** |
| --- | --- | --- | --- | --- |
| **δ^13^C_n_** |  |  |  |  |
| Species + Year + Date sampled + Date sampled:Species | 226.8 | 0.00 | 0.27 | 6 |
| Species + Year + Date sampled + Year:Species | 227.0 | 0.19 | 0.24 | 6 |
| **Species + Year + Date sampled + Date sampled:Species + Year:Species** | **227.7** | **0.97** | **0.16** | **7** |
| **δ^15^N** |  |  |  |  |
| Species + Year + Date sampled | 214.0 | 0.00 | 0.28 | 5 |
| **Species * Year * Date sampled** | **214.3** | **0.34** | **0.24** | **9** |
